# Haplotype bias detection using pedigree-based transmission simulation: traces of selection that occurred in apple breeding

**DOI:** 10.1093/hr/uhaf349

**Published:** 2025-12-26

**Authors:** Hideto Mochizuki, Mai F Minamikawa, Kosuke Hamazaki, Miyuki Kunihisa, Shigeki Moriya, Koji Noshita, Takeshi Hayashi, Yuichi Katayose, Toshiya Yamamoto, Hiroyoshi Iwata

**Affiliations:** Laboratory of Biometry and Bioinformatics, Department of Agricultural and Environmental Biology, Graduate School of Agricultural and Life Sciences, The University of Tokyo, 1-1-1 Yayoi, Bunkyo, Tokyo 113-8657, Japan; Institute for Advanced Academic Research (IAAR), Chiba University, 1-33 Yayoi, Inage, Chiba, Chiba 263-8522, Japan; Molecular Informatics Team, RIKEN Center for Advanced Intelligence Project (AIP), RIKEN, 178-4-4 Wakashiba, Kashiwa, Chiba 277-0871, Japan; Institute of Fruit Tree and Tea Science, National Agriculture and Food Research Organization (NARO), 2-1 Fujimoto, Tsukuba, Ibaraki 305-8605, Japan; Institute of Fruit Tree and Tea Science, NARO, 92-24 Nabeyashiki, Shimokuriyagawa, Morioka, Iwate 020-0123, Japan; Department of Biology, Kyushu University, 744 Motooka, Nishi-ku, Fukuoka 819-0395, Japan; Laboratory of Biometry and Bioinformatics, Department of Agricultural and Environmental Biology, Graduate School of Agricultural and Life Sciences, The University of Tokyo, 1-1-1 Yayoi, Bunkyo, Tokyo 113-8657, Japan; Institute of Crop Science, National Agriculture and Food Research Organization (NARO), 2-1-2 Kannondai, Tsukuba, Ibaraki 305-8518, Japan; Institute of Fruit Tree and Tea Science, National Agriculture and Food Research Organization (NARO), 2-1 Fujimoto, Tsukuba, Ibaraki 305-8605, Japan; Laboratory of Biometry and Bioinformatics, Department of Agricultural and Environmental Biology, Graduate School of Agricultural and Life Sciences, The University of Tokyo, 1-1-1 Yayoi, Bunkyo, Tokyo 113-8657, Japan

## Abstract

Breeding perennial fruit trees like apple is constrained by long generation times and limited population sizes, which often lead to repeated use of a few elite cultivars and consequently narrow genetic diversity. To better understand how such selection processes have shaped the current genetic structure, we applied gene-drop simulations—a pedigree-based method using known parentage and genetic maps—to a curated set of 185 apple cultivars used in Japanese breeding programs, genotyped with 11 786 genome-wide single nucleotide polymorphism markers. This approach enabled us to quantify the expected distribution of founder haplotypes and identify genomic regions where observed founder haplotype frequencies significantly deviated from expectation, suggesting potential selection. Notably, biased regions overlapped with loci associated with key fruit traits, such as fructose content, exemplified by an increase in haplotypes from “Golden Delicious.” Furthermore, Gene Ontology analysis revealed enrichment for regions containing genes involved in stress-related and developmental functions, pointing to broader physiological traits under selection. Unlike traditional methods requiring phenotype data, our approach does not depend on trait measurements and can thus uncover cryptic selection signals, including traits that were not explicitly targeted during breeding. This method offers a framework for identifying overlooked genetic regions and underutilized founder alleles, which can be reintroduced to broaden the genetic base and improve breeding outcomes. Furthermore, the approach is adaptable to other perennial crops with available pedigree and genomic data. Our findings demonstrate the power of integrating pedigree structure with genomic information to reveal both historical and ongoing selection in structured breeding populations.

## Introduction

Historical breeding materials are of paramount importance for perennial plants, such as fruit trees, as they have accumulated rich genetic variation over many years [1]. Woody fruit trees, such as apples, are typically clonally propagated and exhibit high heterozygosity, long juvenile periods, and extended generation times. These characteristics, together with the retention of pedigree ancestors through clonal propagation and the frequent maintenance of detailed breeding records, have contributed to the availability of well-documented pedigrees in apple breeding programs. In contrast, in annual crops, such as rice, which are predominantly bred through selfing [2, 3], the structure of pedigrees and the way breeding records are maintained differ substantially. While pedigree information can be recorded in both self-compatible and self-incompatible species, reproductive and propagation strategies substantially influence the feasibility and informativeness of pedigree-based analysis. In perennial clonally propagated crops such as apple, data-driven breeding strategies that incorporate pedigree information have been shown to improve the accuracy of selection and facilitate the identification and utilization of beneficial traits [4].

In recent years, advancements in sequencing technology have made genome-wide genotyping faster and more affordable, broadening its application to plant breeding [5]. For example, genome-wide association studies (GWAS) [6], which identify candidate genes responsible for traits, such as disease resistance, have been applied to various plant species, including apple [7–9], rice [10], wheat [11], maize [12], and citrus [13]. These findings reflect the increasing role of GWAS as a mainstream approach in modern plant breeding, particularly for identifying loci associated with important agronomic traits. Furthermore, in breeding materials with complete pedigree and single-nucleotide polymorphism (SNP) information, founder haplotypes can be visualized and automatically traced [7], enabling breeders to assess how ancestral genetic contributions have changed over generations.

Gene-drop simulation is a powerful approach for assessing genetic contributions in managed populations [14], particularly within breeding programs. It is a pedigree-based Monte Carlo simulation that models the inheritance of alleles from founder individuals through generations according to Mendelian rules. By repeatedly simulating this process, the expected distribution of alleles or haplotypes under neutral inheritance can be estimated, allowing for comparison with observed data to detect deviations that may indicate selection or drift. Initially developed as a theoretical framework to quantify founder contributions and genetic diversity [14], it was later implemented in pedigree-based genetic analysis tools [15] and applied to practical breeding contexts, such as evaluating ancestral contributions and allele survival in livestock populations [16]. Several studies have demonstrated its utility in modeling gene flow and understanding inbreeding dynamics in controlled populations [14, 15]. Minamikawa *et al.* [7] employed gene-drop simulations in apple breeding to validate the non-random transmission of founder haplotypes identified through GWAS, revealing that certain haplotypes associated with traits, such as skin color, were transmitted more frequently than expected under Mendelian inheritance. For the analyses presented here, we define the “whole founder haplotype” as the set of haplotypes across all chromosomes inherited from a founder, distinguishing it from the locus-specific use of the term “haplotype.” This definition is used in the genome-wide gene-drop simulation conducted in the present study. While gene-drop simulations have been widely applied in livestock and other animal breeding systems to study ancestral contributions and allele survival, the study of Minamikawa *et al.* was among the first to adapt this approach to perennial fruit crops, thereby demonstrating its utility in crops like apple with well-documented pedigrees. In contrast, livestock studies have primarily focused on estimating retention probabilities across generations [16] or modeling gene flow in pedigree-based conservation schemes [15]. While these studies underscore the versatility of gene-drop simulation, they have not explored region-specific signals of selection at SNP-level resolution. Importantly, to our knowledge, no study to date has integrated this approach with genome-wide SNP data to identify local deviations indicative of selection, a methodological gap addressed in this study.

Building on the work of Minamikawa *et al.* [7], this study aimed to identify genomic regions that might have been subjected to selection during the breeding process by detecting biases in founder haplotype transmission using genome-wide gene-drop simulations. In apple breeding, cultivars such as Fuji have made substantial contributions to the pedigrees of modern cultivars, potentially introducing haplotype frequency biases that obscure signals of historical selection. Therefore, a method that accounts for such contribution-driven biases is essential for accurately identifying genomic regions under selection. Additionally, by visualizing pedigree structures using Helium [17] and applying gene-drop simulations across generations, we aimed to investigate both the transmission biases of the founder haplotypes and potential causes, such as historical selection pressures.

## Results

### Detecting frequency bias of founder haplotypes using gene-drop simulation

**Figure 1:**
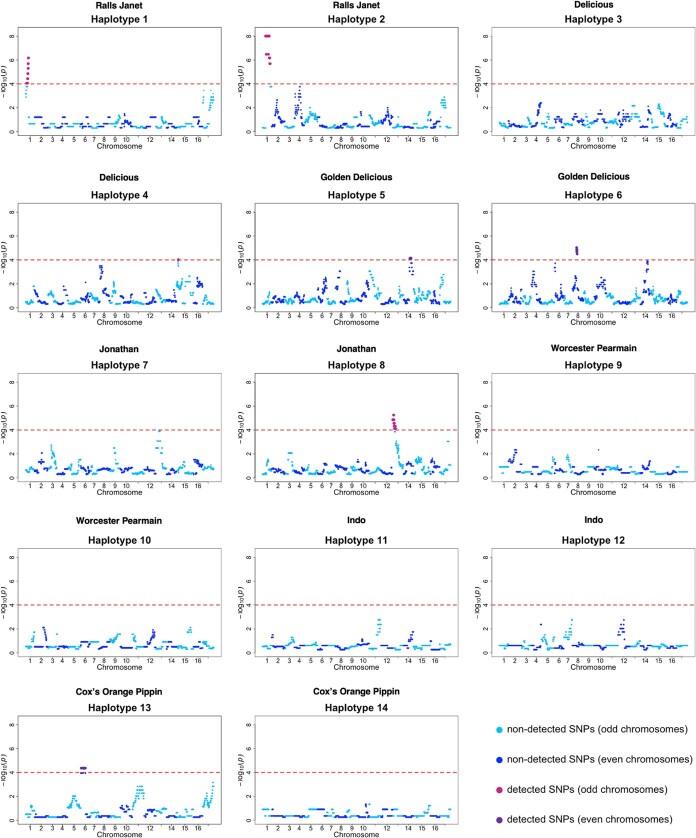
Detection of frequency bias in the 14 whole founder haplotypes using gene-drop simulations. Regions considered to be significantly selected for each whole founder haplotype are shown. The horizontal axis represents the genomic position of SNPs, and the vertical axis shows the $-{\log}_{10}(p)$ value from the simulations. Purple dots indicate significant regions, while blue dots represent non-significant regions. The red dashed lines mark the threshold ($-{\log}_{10}(p)=4$).

A frequency bias was identified for seven whole founder haplotypes (1, 2, 4, 5, 6, 8, and 13) at specific SNPs across the genome, when the entire population was tested (Fig. 1; Table S1). These biases reflect deviations from the expected haplotype frequencies assigned to each SNP locus under Mendelian inheritance. In the tests for individual generations, significant changes in the frequencies of some founder haplotypes were observed starting from the third generation (Figs S2–S5). The SNPs at which founder haplotype frequencies changed significantly in the separate-generation tests were identical to those detected as significant in the analysis of the entire population. Hereafter, the results will focus on the population-wide test, which was considered to have higher statistical power.

### Concordance between SNP markers with biased frequencies of founder haplotypes and previously reported GWAS peaks or reference annotation data

The SNP markers with significantly altered founder haplotype frequencies coincided with the GWAS peaks for 11 fruit traits (Table S1). Among the 11 blocks with significantly altered founder haplotype frequencies, six showed changes that were consistent with the expected phenotypic changes; that is, the direction of the haplotype frequency change matched the direction of the trait change (e.g., an increase in a haplotype that has positive effect alongside an increase in phenotypic value) (Figs S6, S8–S11, and  S14). In contrast, three blocks did not exhibit consistency, and two blocks displayed no clear phenotypic trends across generations (Figs S7, S12--S13, and S15–S16). For example, in one consistent case, the phenotypic value of malic acid decreased over time, the frequency of the associated founder haplotype increased significantly, indicating a correlation between haplotype frequency and phenotypic value (Figs S9–S11). Furthermore, for other traits, the frequencies of haplotypes 1 and 2, derived from “Ralls Janet,” increased at SNPs near markers associated with Degree of Mealiness [18], a trait related to fruit firmness and storability.

To explore the potential biological basis of these associations, we identified 68 SNP markers included in 41 genes within genomic regions corresponding to founder haplotypes previously associated with GWAS peaks (Table S1). The genes closely linked to these SNP markers are implicated in biological processes (BPs) beyond fruit traits, including cell division, pollen development and germination, drought and salt stress tolerance, reproduction, and maintenance of vital activities. In addition, among the SNP markers with significantly biased founder haplotype frequencies that did not correspond to known GWAS peaks, 812 SNPs overlap with 331 gene regions (Table S1).

### Gene ontology enrichment analysis to estimate the reasons for selected regions

**Figure 2:**
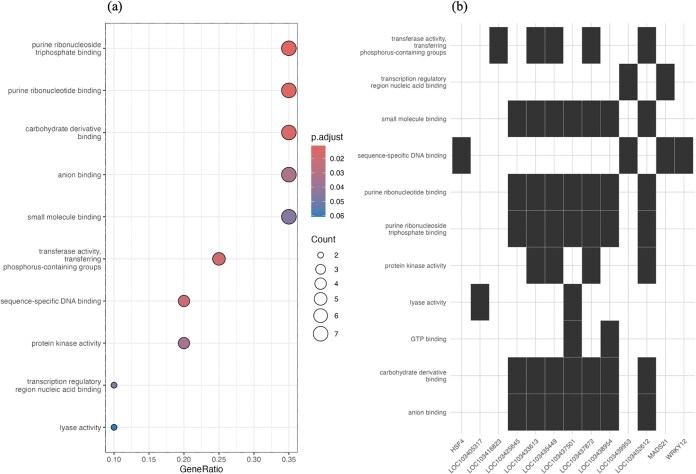
Results of Gene Ontology (GO) enrichment analysis in MF. (a) The GO terms within the MF category enriched in genes containing SNP markers with significantly altered founder haplotype frequencies. The horizontal axis represents the proportion of genes associated with each GO term, and the size of each dot represents the number of genes. The color of the dots indicates the *P* value, with red representing statistically significant associations, implying a stronger likelihood of being linked to that function. (b) A figure showing the genes associated with each GO term. Sequence-specific DNA binding (fourth row from the top), which had the lowest *P* value, was linked to four genes: *HSF4*, *LOC103439953*, *MADS21*, and *WRKY12*.

Gene ontology (GO) enrichment analysis [19] revealed that, under a significance threshold of *P* < 0.05, no GO terms in the BP and cellular component (CC) category were significantly enriched among genes containing the 812 SNP markers located within founder haplotypes that showed significantly altered frequencies and unmatched GWAS peaks. GO terms of the molecular function (MF) category, “transferase activity, transferring phosphorus-containing groups” and “sequence-specific DNA binding,” were significantly (*P* < 0.05) enriched within the genes (Fig. 2a). “Sequence-specific DNA binding” had the lowest *P* value in the MF category. Additionally, when we analyzed the genes associated with each function shown in Fig. 2a, *HSF4*, *LOC103439953*, and *WRKY12* genes were each linked to one function (Fig. 2b).

## Discussion

### Causes and implications of founder haplotype frequency changes

More than half of the genomic blocks identified in this study displayed patterns consistent with the expected outcomes based on previously reported founder haplotype effects [7]. A high-profile example was the increase in founder haplotype 6 (from “Golden Delicious”) at the region around SNP_FB_1117728 on chromosome 8, which is associated with increased fructose content and showed a significant increase in frequency across generations (Fig. S6). This suggests that the haplotype at the SNP_FB_1117728 may have been selected to enhance fructose levels. Given that fructose is sweeter than other sugars [20] and that consumers often prefer sweeter fruit cultivars [21], it is plausible that the pursuit of fruit qualities favored by consumers has resulted in selection of apples with higher fructose content by breeders. Similarly, haplotype 2 from “Ralls Janet,” associated with firmer texture, also increased in frequency at the region shown in Fig. S9 and corresponding to markers reported [18], aligning with breeding targets such as improved fruit texture, including firmness. These selected haplotypes originate from founder cultivars historically recognized for their breeding value. The observed patterns are consistent with Fig. S11, and we note that sweetness perception is influenced not only by fructose content but also by relative acidity [22]. Therefore, the potential contribution of acidity QTL to perceived sweetness should also be considered. For example, “Golden Delicious” has long been extensively used as a crossing parent in breeding programs worldwide due to its genetic ability to produce offspring with improved sweetness, storability even though breeders at the time did not quantify this genetic potential. Likewise, “Ralls Janet” is known for its firm flesh, which has been introgressed into many modern cultivars. The persistence of such founders' haplotype may reflect their consistent performance under diverse environmental and market demands. Founder haplotypes 1 and 2 from “Ralls Janet” also increased in frequency on chromosome 1. Apple scab, one of the most serious diseases in apple, can be genetically controlled by major resistance genes, such as *Rvi6*, located on this chromosome. Although the apple breeding program at National Agriculture and Food Research Organization (NARO) perform seedling-stage screening for scab resistance—using DNA markers or spore inoculation—when progeny is derived from parents carrying major resistance genes, these haplotypes do not carry *Rvi6*. Therefore, their increased frequency on chromosome 1 is unlikely to result from selection for apple scab resistance in the initial selection. While many haplotype frequency changes can be explained by selection for known, targeted traits, not all patterns follow this rule. In several instances (Fig. S8), founder haplotypes that underwent substantial frequency changes may have been favored due to loci linked to unmeasured or indirectly selected traits. Many important traits in fruit breeding, such as sugar content, firmness, and acidity, are complex and polygenic, often involving numerous small-effect loci that remain undetected in traditional Quantitative Trait Locus or GWAS analyses but can still respond to selection if cumulatively beneficial. Some founder haplotypes may have been retained due to selection on unmeasured fruit quality or agronomic attributes. Similarly, founders, such as “Delicious” and “Ralls Janet,” despite their susceptibility to certain diseases such as apple scab, have been used as parents for their fruit quality and adaptability. These cases illustrate that both intentional selection and indirect selection driven by environmental or regional preferences can shape founder haplotype frequencies in ways not attributable to explicit trait-based targets. Previous studies have applied selection sweep analyses in apples to identify genomic regions under selection, particularly in the context of domestication or long-term evolutionary change. For example, Liao *et al.* [23] analyzed selection signals across a wide range of cultivated and wild apple accessions, while Skytte af Sätra *et al.* [24] focused on patterns of variation within cultivated apple accessions. In contrast, our study introduces a pedigree-based gene-drop simulation approach to detect more recent, breeding-program-specific selection patterns in a structured population. While some of the regions we identified overlap with those from previous studies, others appear to be unique, reflecting program-specific or environmentally influenced selection. Thus, our approach complements existing methods by capturing both intentional and unintended selection outcomes within modern breeding programs.

### Gene ontology insights and potential linked selection

**Figure 3:**
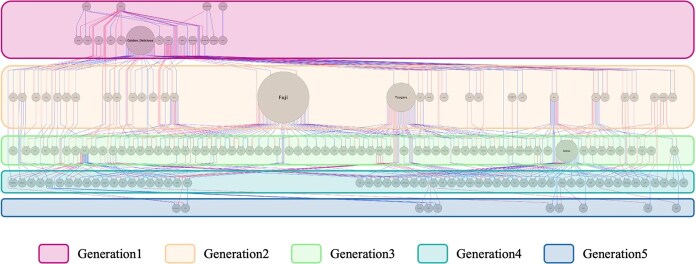
Generational partitioning of apple cultivars based on the pedigree chart drawn using Helium. This diagram shows the generations and contributions of 185 Japanese cultivars. Each node represents a cultivar, and the dark grey nodes indicate uninformed parent individuals. Each row shows the partitioned generation using the pedigree visualization tool Helium. The node sizes indicate their contributions as parents in subsequent generations. The large node representing “Fuji” reflects its significant role in breeding, as “Fuji” was used extensively for mating and has the largest contribution to subsequent generations. The pedigrees used are described in Table S3.

The GO enrichment analysis revealed several enriched terms in the MF category, including transferase activity, transfer of phosphorus-containing groups, and sequence-specific DNA binding (Fig. 2). Notably, the term “sequence-specific DNA binding” includes four genes—*HSF4*, *LOC103439953*, *MADS21*, and *WRKY12*—that are all located on chromosome 1, within regions showing significant shifts in founder haplotype frequencies. These genes co-localize on chromosome 1, which also harbors other loci with increased founder haplotype frequencies in our analysis, raising the possibility of linked selection. Notably, Skytte af Sätra *et al.* [24] (Fig. 3) detected selection signature near the beginning of chromosome 1, around 28.9 to 29.9 Mb, in cultivars adapted to northern climates, providing a point of comparison with our findings. Among the genes in this region heat shock factors (*HSFs*) are of particular interest because they are involved in flavonoid biosynthesis and drought resistance [25], and in bananas, *HSF4* is regulated by cold stress [26], suggesting its potential involvement in both heat and cold tolerance in apples. *MADS21* contributes unsaturated fatty acid metabolism in palm trees [27] and is involved in fat development and flowering transition in *Arabidopsis* [28–30]. Members of the *WRKY* gene family are well known for their role in abiotic stress responses, with some also contributing to biotic stress responses, such as resistance to *Alternaria alternata*, a fungal disease in apple [31]. Interestingly, *WRKY12* exhibits opposite effects on flowering under short-day conditions [32]. The co-occurrence of these functionally diverse, stress-related genes in a chromosome 1 region showing haplotype frequency bias suggests that breeding programs may have inadvertently favored founder haplotypes that confer broad adaptability—potentially including abiotic and biotic stress tolerance—despite not directly targeting these traits. The genome-wide gene-drop simulation employed in this study highlights how unmeasured or implicit phenotypes, such as cold tolerance in northern regions, can influence haplotype frequencies and breeding outcomes, even in the absence of direct phenotypic selection. It should be noted that the *S* locus of self-incompatibility (SI) on chromosome 17, while critical for preventing crosses between individuals with identical S-alleles, is not a direct target of artificial selection in apple breeding. Instead, *S* alleles are typically maintained by balancing selection to preserve cross-compatibility within breeding populations. This contrasts with traits, such as sugar content, which tend to accumulate through directional selection. Chromosome 17 also contains genes significantly associated with glucose, fructose, and sucrose content, as identified by GWAS. However, unmeasured traits or linkage with undesirable alleles—including certain S-alleles—may influence haplotype frequency dynamics in this region. For example, accumulation of *S*-alleles may restrict future crossing possibilities, posing potential challenges for breeding. Furthermore, not all genomic regions identified by GWAS exhibited clear selection signals in our gene-drop simulations, as the latter captures historical selection pressures over pedigrees rather than single-trait associations. The complex linkage relationships and balancing selection at the SI locus may thus obscure or complicate detection of significant frequency deviations.

## Conclusions and prospects for broader use

Our approach provides a comprehensive approach for identifying genomic regions potentially associated with disease resistance in apples and other fruit trees. Serious diseases, such as Alternaria blotch [33] and fire blight, continue to impact apple breeding, with susceptibility in ancestral apple cultivars like “Indo” and “Delicious” [18] inherited by several offspring, and cultivars, such as “Fuji,” also showing susceptibility to bacterial and fungal pathogens [34, 35]. While marker-assisted selection is available for these diseases [35, 36], it is labor-intensive when aiming to evaluate resistance across diverse germplasm. In contrast, our approach can detect regions linked to resistance not only for well-characterized diseases but also understudied yet severe pathogens, opening new avenues for breeding. Furthermore, this method is broadly applicable beyond apples to other fruit tree species, including citrus [37] and peach [38], particularly where pedigree information is limited but founder haplotype data are available. By comparing regions with significantly altered founder haplotype frequencies to annotated regions, we can identify useful genetic regions that may not have been directly targeted by breeding programs. As our method does not require phenotypic measurements, it allows exploration of a wider range of valuable genetic regions. Overall, our pedigree-based gene-drop simulation approach is a valuable tool for detecting biases in founder haplotype frequencies while minimizing the influence of breeding parents with substantial contributions to subsequent generations, such as “Fuji,” thereby providing new opportunities for future breeding efforts.

## Materials and methods

### Plant materials, pedigree, genotypes, and phenotypes

In total, 185 Japanese apples (*Malus domestica* Borkh.) cultivars/selections were used for this study, all of which were cultivated at the Apple Research Station, Institute of Fruit Tree and Tea Science, NARO, Japan [7]. These cultivars mainly originated from seven founder cultivars: “Ralls Janet,” “Delicious,” “Golden Delicious,” “Jonathan,” “Worcester Pearmain,” “Indo,” and “Cox’s Orange Pippin.” Pedigree information for all cultivars was utilized. SNP genotypes were obtained for 11 786 markers using the Illumina Infinium array, and missing SNP data were imputed using Beagle ver. 4.0 [39], as described by Minamikawa *et al.* [7]. The phased SNP data were assigned to 15 founder haplotypes, including 14 derived from the seven founders and one representing a haplotype of unknown origin (haplotype 15). All cultivars used in this study were diploid. Phenotypic data for 27 fruit traits evaluated over multiple years were also used in this study (Table S2).

### Gene-drop simulation to detect the frequency bias of founder haplotypes

We conducted pedigree-based gene-drop simulations [14] to estimate the expected segregation of founder haplotypes under Mendelian inheritance. One of the two founder haplotypes at each SNP locus was inherited with a probability of 1/2 at each meiosis. The simulation was performed across multiple founder haplotypes, and for each locus, the observed haplotype frequencies in the progeny population were compared to the expected frequencies under the null hypothesis of random segregation. The expected frequency distribution of founder haplotypes was modeled using a multinomial distribution as follows:


$$ \mathcal{M}\left(\boldsymbol{x}\mid \boldsymbol{\mathrm{\pi}}, n\right)=\frac{n!}{\Pi_{k=1}^K{x}_k!}{\Pi}_{k=1}^K{\mathrm{\pi}}_k^{x_k} $$

where $\boldsymbol{x}=\left\{{x}_1,{x}_2,\cdots, {x}_k\right\}$ is the number of times the founder haplotype has been obtained, $\boldsymbol{\mathrm{\pi}} =\left\{{\mathrm{\pi}}_1,{\mathrm{\pi}}_2,\cdots, {\mathrm{\pi}}_{\mathrm{k}}\right\}$ where is the probability of obtaining a combination of founder haplotypes, *n* is the number of haplotypes ($2\times$ population size), and *k* is the number of possible combinations of founder haplotypes in the population and $\boldsymbol{\pi}$ and $\mathbf{x}$ satisfy ${\sum}_{k=1}^K{x}_k=n$, ${\sum}_{\pi =1}^K{\pi}_k=1$. Founder haplotypes were manually assigned by Kunihisa *et al.* [18], using phased SNP haplotype information together with documented parent–offspring relationships. This approach enabled unambiguous tracing of haplotypes through the pedigree and allowed us to distinguish haplotypes that were identical-by-state but originated from different founders, thereby avoiding misattribution in genomic regions where founders share identical-by-descent segments. Although it is possible to obtain the frequency of founder haplotypes analytically as described above, it was difficult to do so for all individuals in the apple population in this study, which consisted of 14 founder haplotypes (and all rare non-founder haplotypes binned together as “haplotype 15”) and 185 parental cultivars. To address this, we simulated the spread of founder haplotypes in later generations by creating empirical frequency distributions of founder haplotypes using 10 million gene-drop simulations based on pedigree information. To ensure robustness of results against sampling variance and to reduce false-positives due to uneven founder usage, we tested simulation sizes ranging from 10 000 to 10 million iterations and confirmed that significant marker detection stabilized beyond 500 000 iterations. Thus, 10 million iterations were chosen to minimize Type I and Type II errors. This simulation assumed that founder haplotypes were obtained randomly when breeders did not select them for the null hypothesis. This empirical frequency distribution was used as the null distribution. The *P* value for the observed founder haplotype frequency in each genetic region was defined as the difficulty of occurrence in the simulation (Fig. S1). This method detected SNP markers as regions that may have been selected by setting the threshold to $-{\log}_{10}(p)=4$ and detecting significant changes in the frequency of founder haplotypes when the ${-\log}_{10}(p)$ value exceeds the threshold. In this study, because the focused founder haplotypes were tested using the percentage of all founder haplotypes, the horizontal axis in Fig. S1 corresponds to *k* of the multinomial distribution, and the vertical axis to ${x}_{\mathrm{n}}$. Additionally, we tested for bias within each generation separately, as analyzing the entire population could obscure founder haplotypes that had changed in frequency during earlier generations. The pedigree drawing software Helium was used to separate generations for analysis (Fig. 3 ) [17]. Each row in the diagram corresponds to a partitioned generation. Generation one comprised seven founder cultivars. For later generations, columns fifth and sixth were combined to define Generation five due to few rows corresponding to Generation six. To reduce bias from uneven founder usage, such as the disproportionate influence of highly utilized cultivars like “Fuji,” we compared the observed founder haplotype frequencies to those expected under random segregation. This normalization enabled detection of deviations more likely attributable to selection rather than to pedigree structure or biased mating patterns.

### Comparison of SNP locus, in which the frequency bias of founder haplotypes was detected, with previously reported GWAS peaks

SNP markers located within genomic regions where founder haplotypes frequencies showed significant deviations, as detected by the previous method were compared with previously reported GWAS peaks for fruit traits [7] to determine whether these regions were likely selected based on fruit traits. Specifically, when an SNP located in a region with significantly deviated founder haplotype frequencies overlapped with an SNP marker identified in the GWAS, we tracked the change in the founder haplotype frequency of the marker and the generational trend in the associate phenotypic values. Furthermore, we estimated the effects of each founder haplotype on the marker using BayesB with Markov chain Monte Carlo methods [40, 41]. Marker regression models, such as BayesB, assume that genotypic values $u$ are determined by the linear sum of the marker effects, as follows:


$$ {u}_i={\sum}_{j=1}^J{\mathrm{\gamma}}_j{\sum}_l^{L_j}\left({x}_{ijl}+{x}_{ijl}^{{\prime}}\right){\mathrm{\beta}}_{jl} $$

where *J* represents the total number of markers and ${L}_j$represents the number of founder haplotypes at the *j*-th marker. The variable ${x}_{ijl}$ (${x}_{ijl}^{{\prime}}$) denotes the maternal (paternal) haplotype of marker *j* for cultivar *i* and equals to 1 if the maternal (paternal) haplotype is the *l*-th haplotype ($l=1,2,\cdots, {L}_j$) and 0 otherwise. The parameter ${\mathrm{\gamma}}_j$ indicates the posterior probability of the *j*-th marker having a quantitative trait locus, while ${\mathrm{\beta}}_{jl}$ represents the genetic effect associated with the *l*-th founder haplotype at marker *j*. The effect ${\mathrm{\beta}}_{jl}$ is assumed to follow a Gaussian distribution $\mathcal{N}\left(0,{\mathrm{\sigma}}_{\mathrm{\beta}_j}^2\right)$, as described by Minamikawa *et al.* [7]. This study identified the combinations of SNP markers and founder haplotypes that exhibited significant increase or decrease in frequency. The markers were compared with the GWAS peaks for fruit traits, and the set of markers that may have undergone selection based on fruit traits was narrowed. We then tracked the frequency of founder haplotype in each generation, as divided by Helium, and combined this information with the founder haplotype effect ${\mathrm{\beta}}_{jl}$, to investigated whether the markers and founder haplotypes were strongly associated with selection on fruit traits.

### Inferring reasons for shifts in founder haplotype frequency via gene set enrichment analysis

The methods described above identified SNP markers located in genomic regions where specific founder haplotypes exhibited significant frequency shifts. To explore whether these regions were associated with fruit traits, we compared the identified SNPs with those detected by GWAS. Therefore, we investigated SNP markers associated with such traits. First, we downloaded the gene regions assigned to the “Golden Delicious” doubled-haploid tree reference and their annotations from Phytozome database [42]. Using the 812 SNP markers detected with the above method, we extracted the gene regions containing these markers. The extracted 331 genes were then subjected to gene set enrichment analysis using the R package “ClusterProfiler” [43]. The GO terms of these genes were analyzed in three categories: BP, MF, and CC.

## Supplementary Material

Web_Material_uhaf349

## Data Availability

The genotype and phenotype data used in this study are publicly available from Minamikawa *et al.* (2021) [DOI: 10.1038/s41438-021-00485-3], which provides access to the DNA marker and trait datasets used for Japanese apple breeding. No new datasets were generated during the current study. Information on DNA markers is available from Supplementary Data Table.
