# Supplementary material for: Haplotype bias detection using pedigree-based transmission simulation: traces of selection that occurred in apple breeding
Source: Hortic Res. 2025 Dec 26;13(3):uhaf349. doi: 10.1093/hr/uhaf349 (PMC12977171; doi:10.1093/hr/uhaf349)
Supplement: Web_Material_uhaf349 [file web_material_uhaf349.zip › Supplementary_Figure.pptx]

## Slide 1
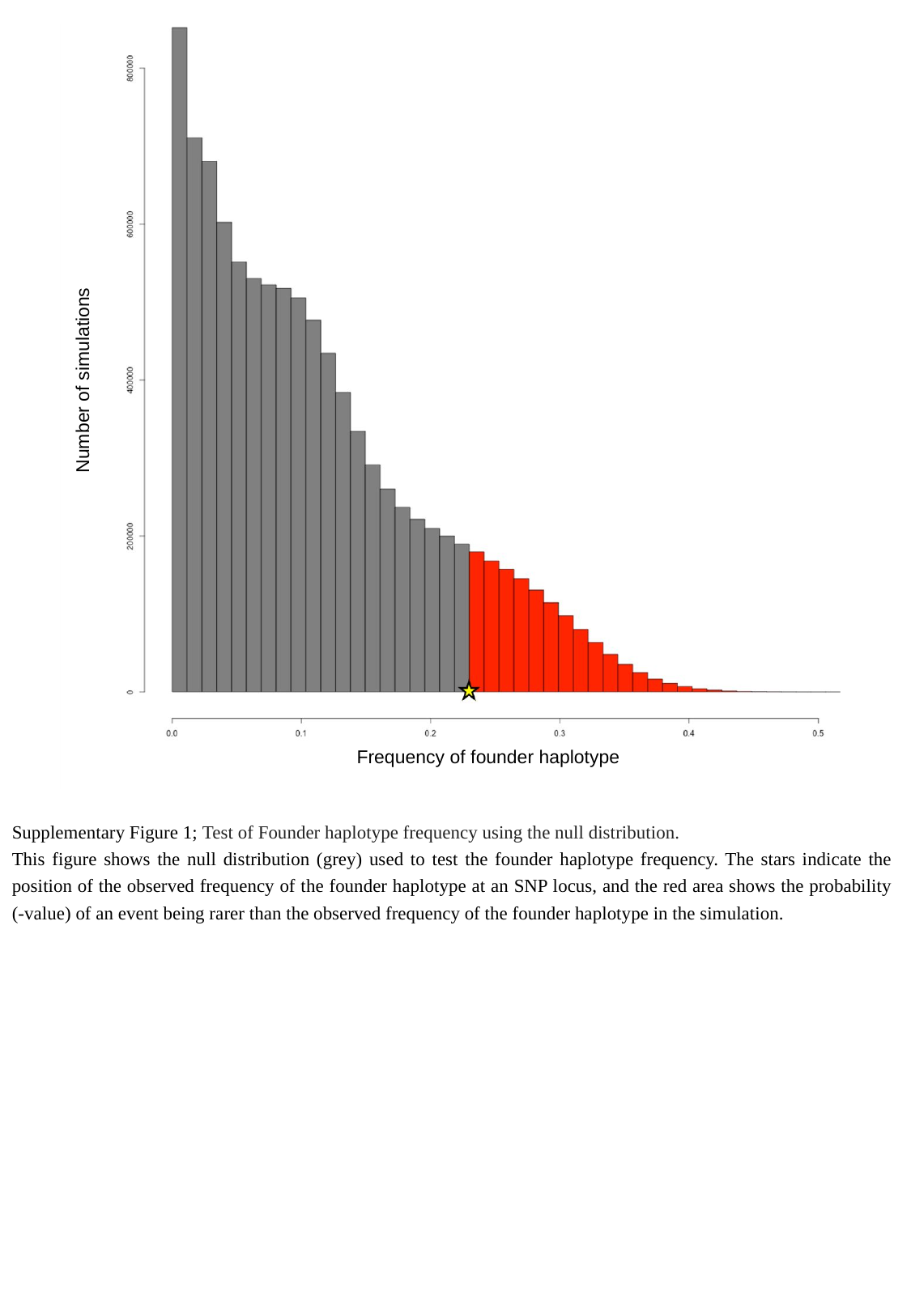

Number of simulations
Frequency of founder haplotype

## Slide 2
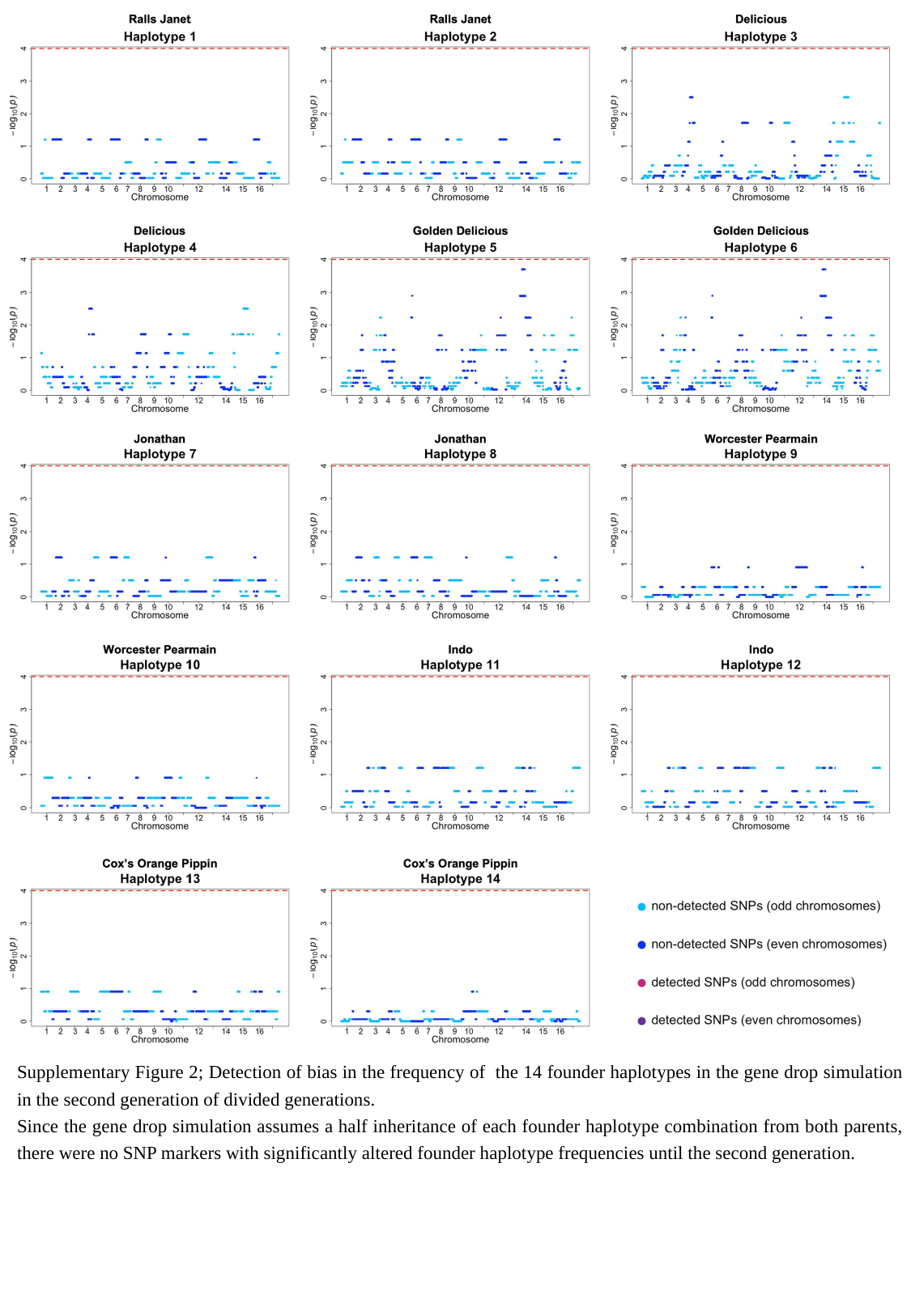

Supplementary Figure 2; Detection of bias in the frequency of the 14 founder haplotypes in the gene drop simulation in the second generation of divided generations.
Since the gene drop simulation assumes a half inheritance of each founder haplotype combination from both parents, there were no SNP markers with significantly altered founder haplotype frequencies until the second generation.

## Slide 3
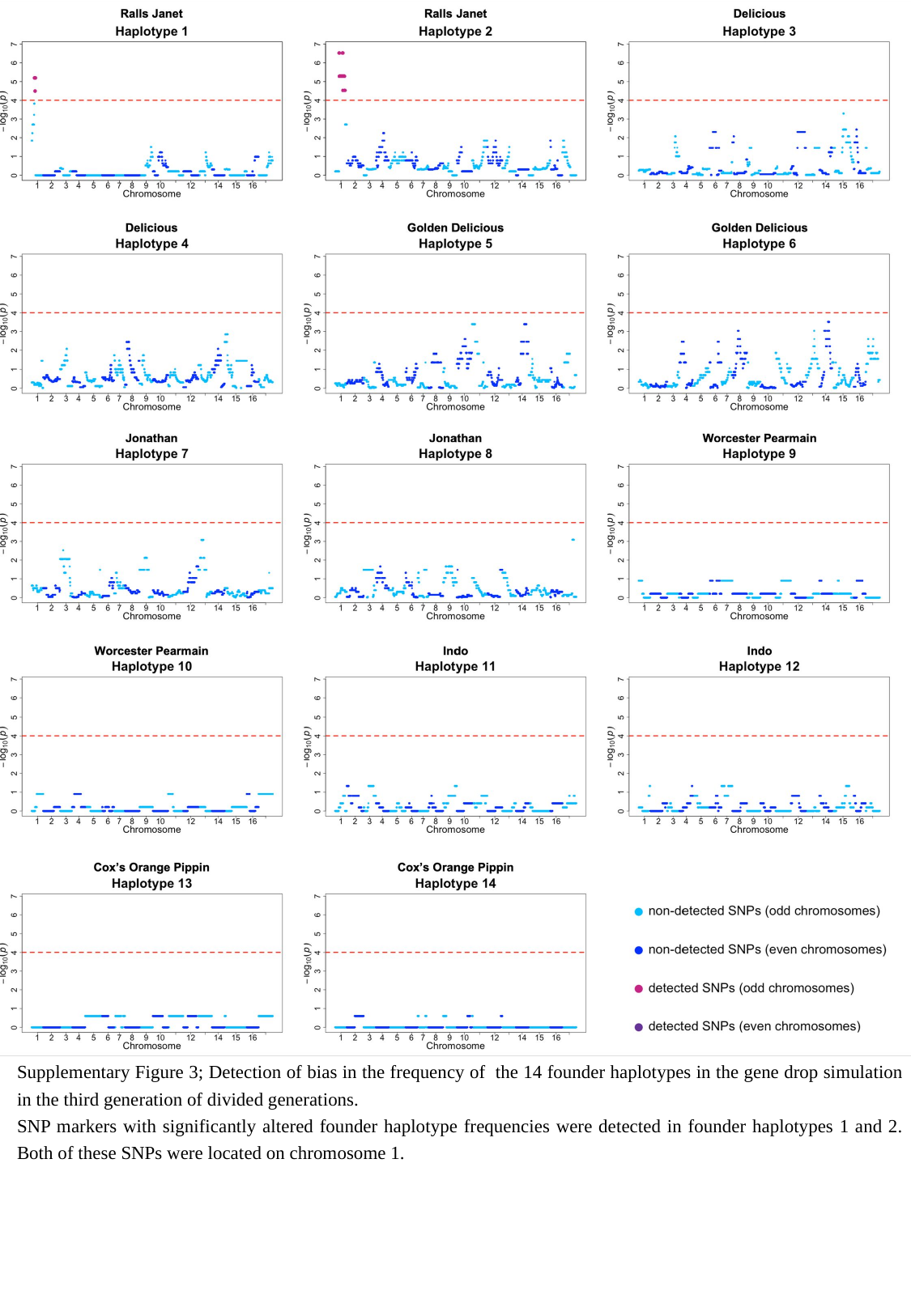

Supplementary Figure 3; Detection of bias in the frequency of the 14 founder haplotypes in the gene drop simulation in the third generation of divided generations.
SNP markers with significantly altered founder haplotype frequencies were detected in founder haplotypes 1 and 2. Both of these SNPs were located on chromosome 1.

## Slide 4
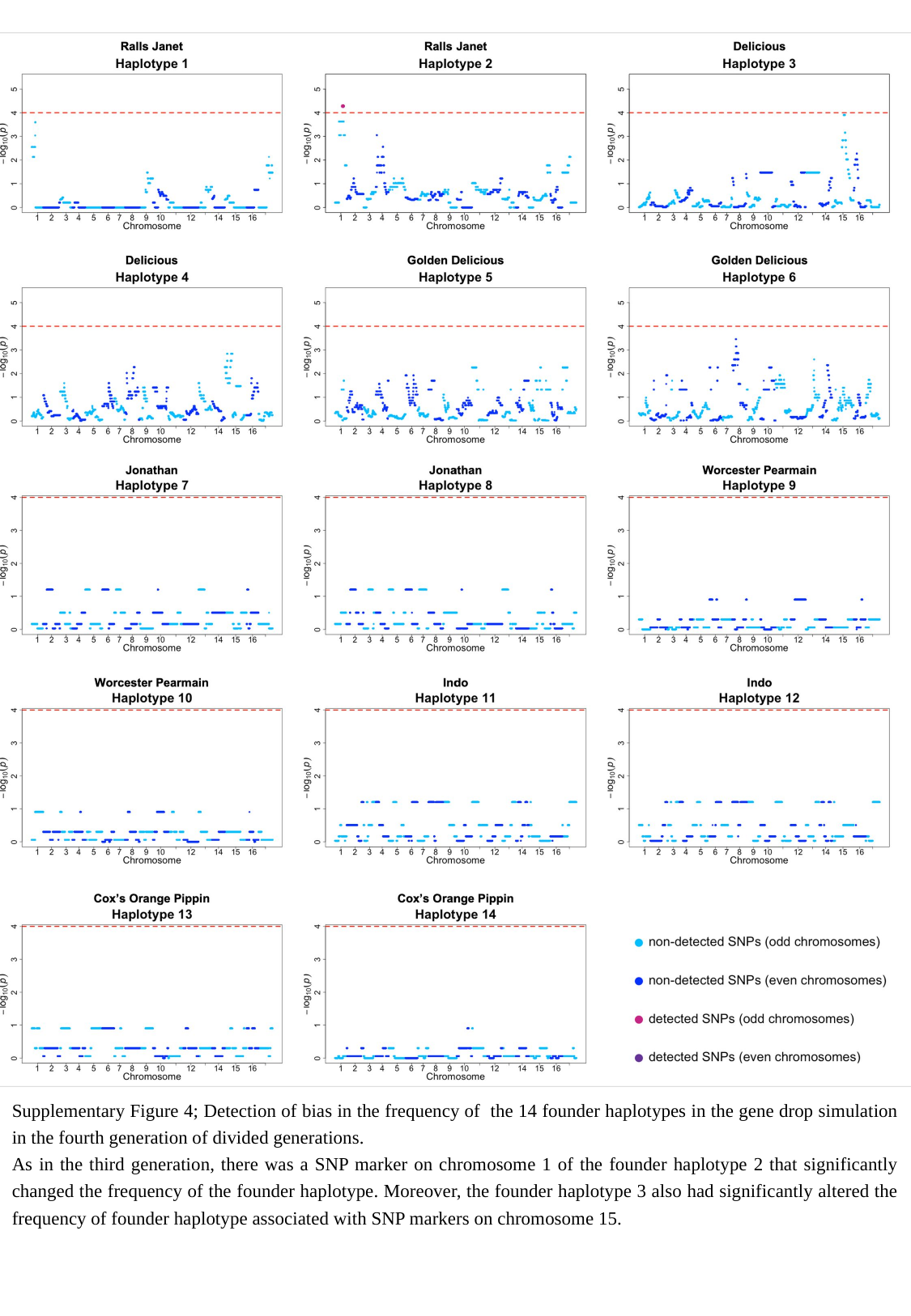

Supplementary Figure 4; Detection of bias in the frequency of the 14 founder haplotypes in the gene drop simulation in the fourth generation of divided generations.
As in the third generation, there was a SNP marker on chromosome 1 of the founder haplotype 2 that significantly changed the frequency of the founder haplotype. Moreover, the founder haplotype 3 also had significantly altered the frequency of founder haplotype associated with SNP markers on chromosome 15.

## Slide 5
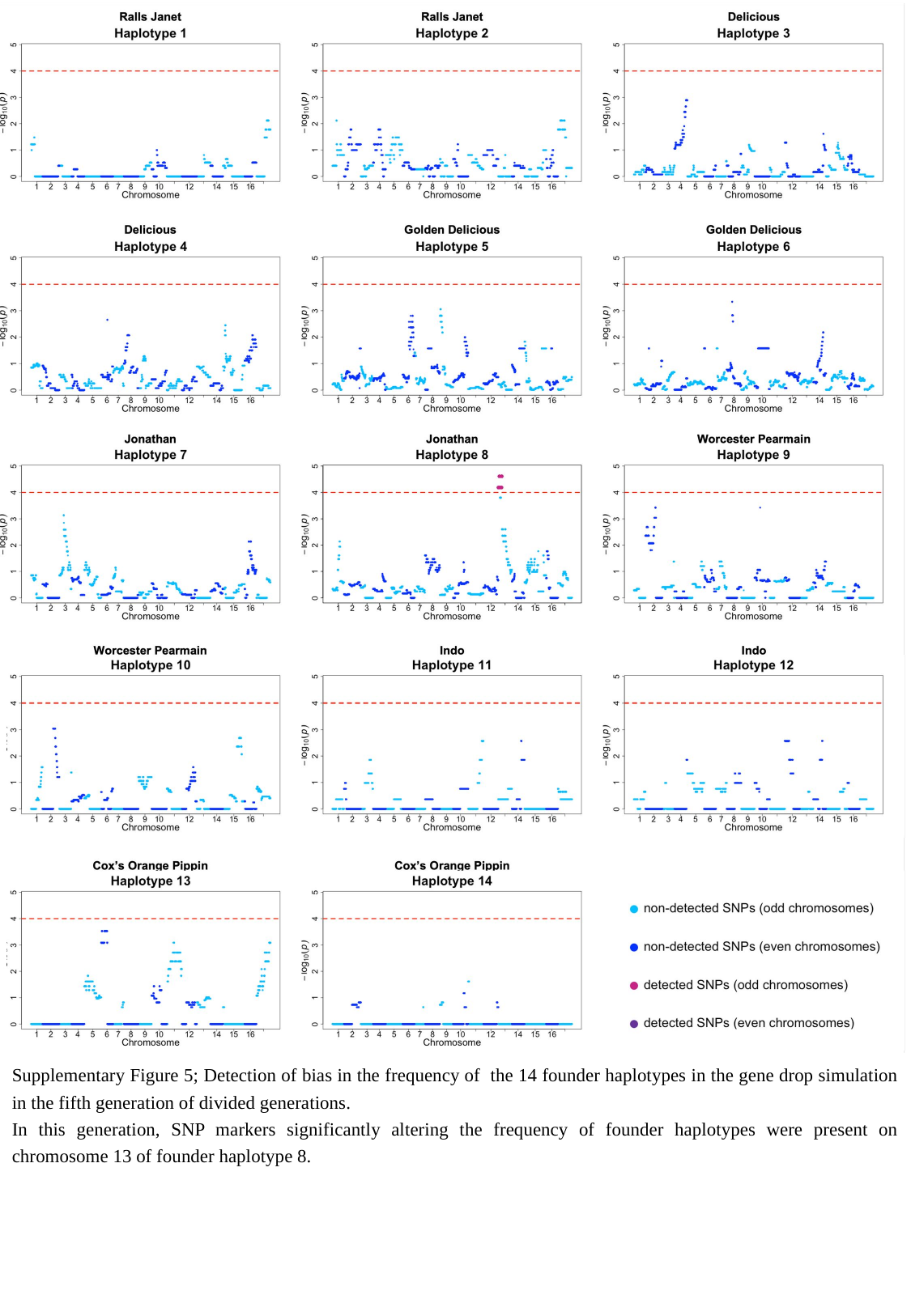

Supplementary Figure 5; Detection of bias in the frequency of the 14 founder haplotypes in the gene drop simulation in the fifth generation of divided generations.
In this generation, SNP markers significantly altering the frequency of founder haplotypes were present on chromosome 13 of founder haplotype 8.

## Slide 6
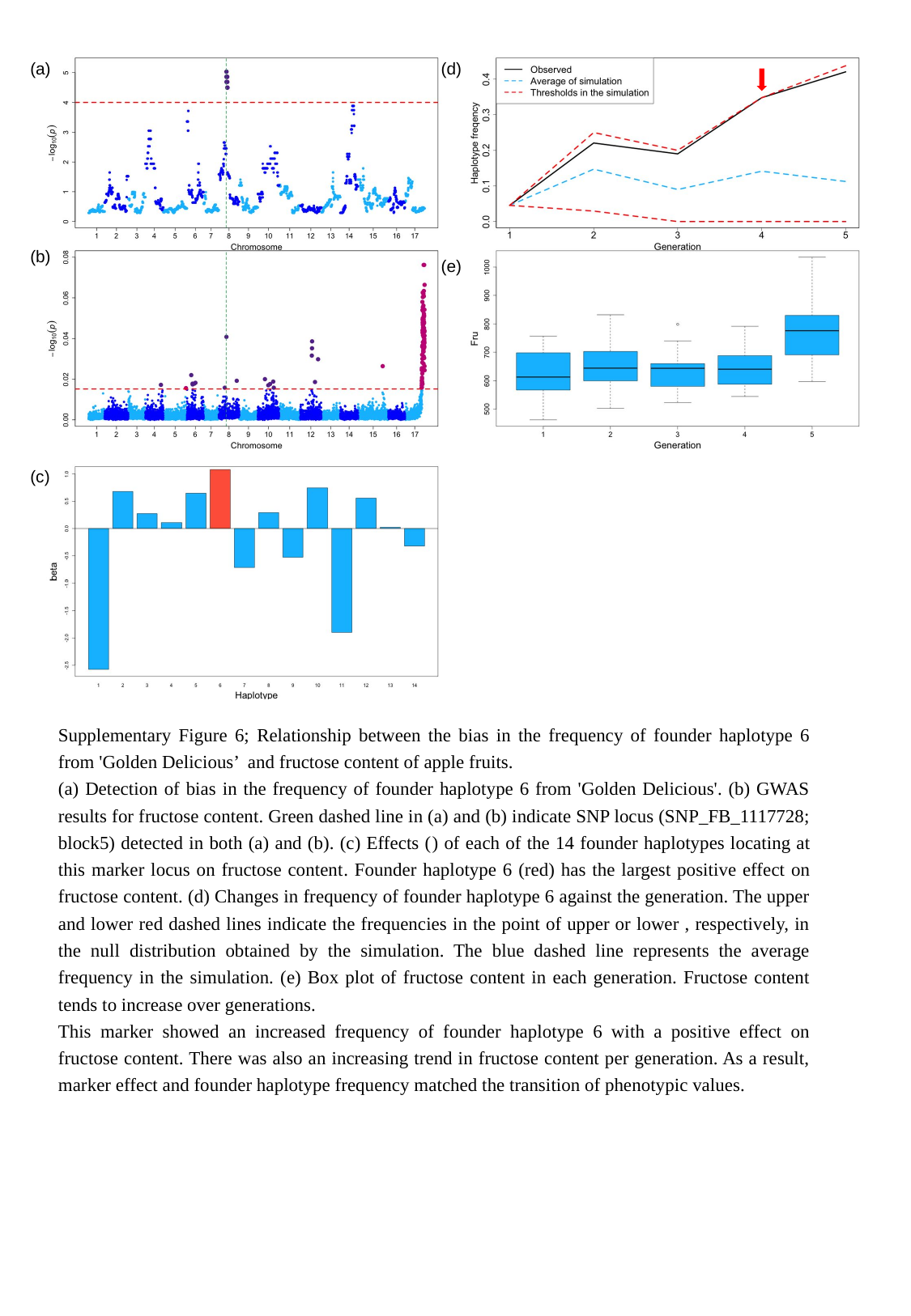

(d)
(a)
(b)
(e)
(c)

## Slide 7
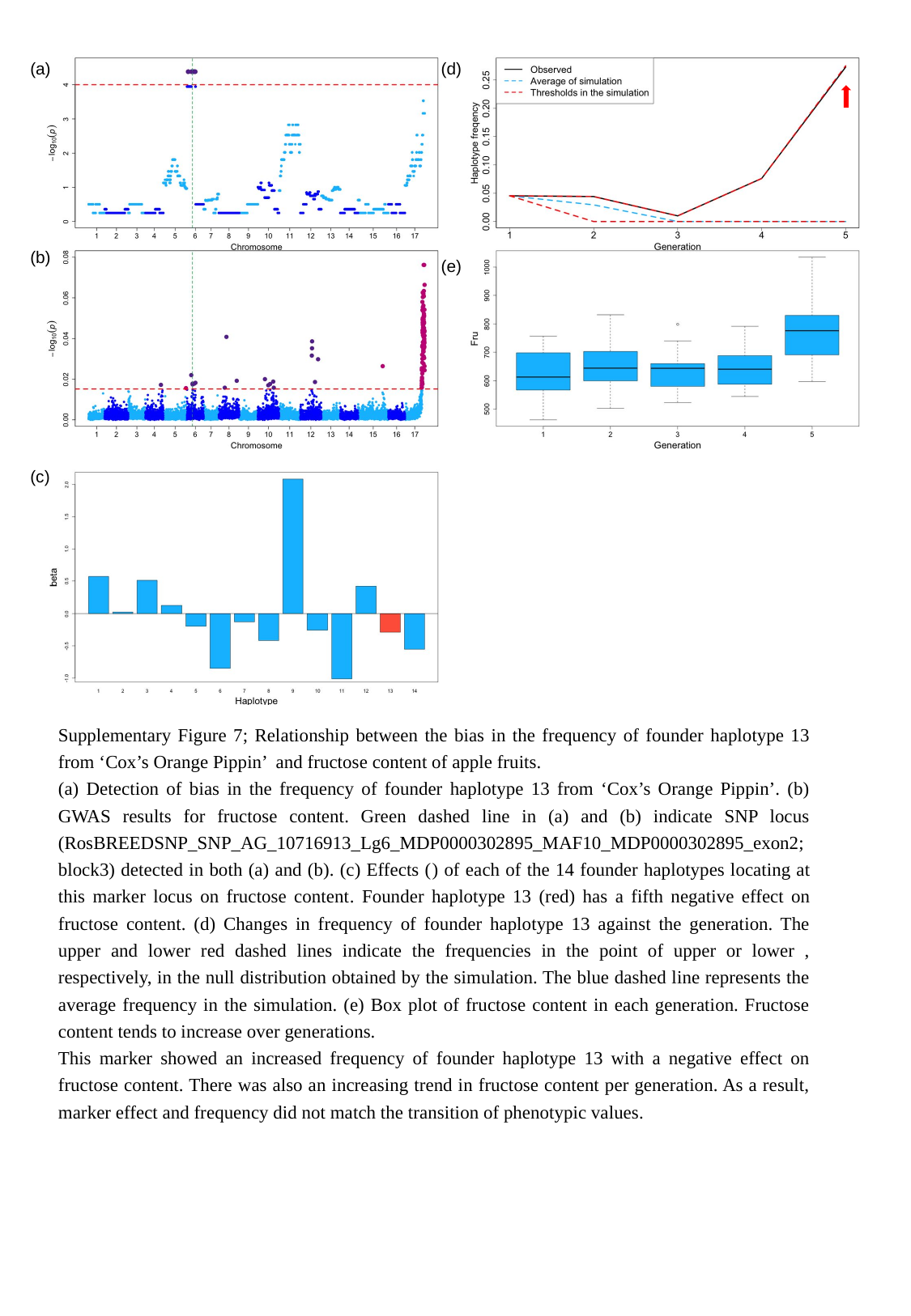

(d)
(a)
(b)
(e)
(c)

## Slide 8
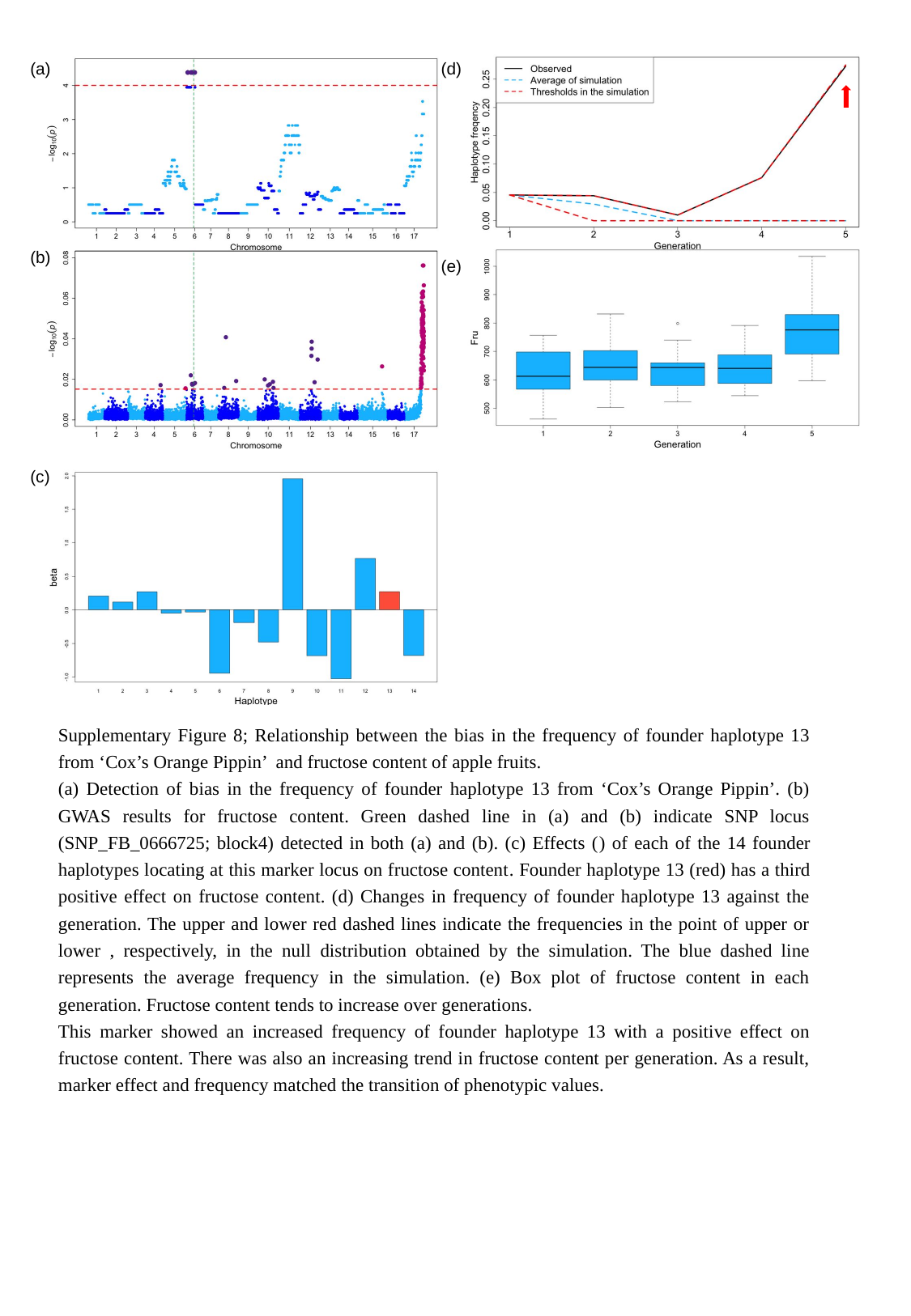

(d)
(a)
(b)
(e)
(c)

## Slide 9
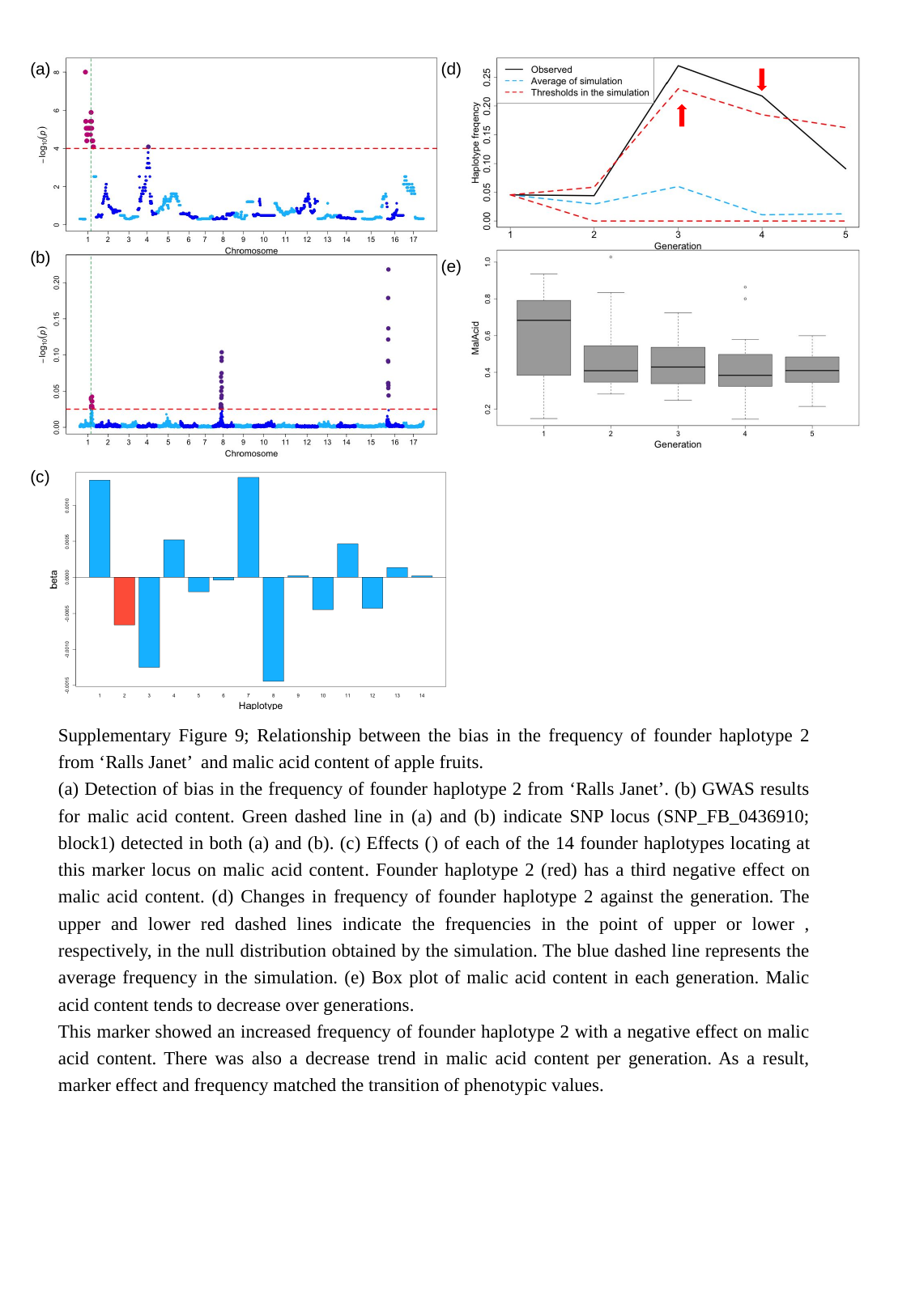

(d)
(a)
(b)
(e)
(c)

## Slide 10
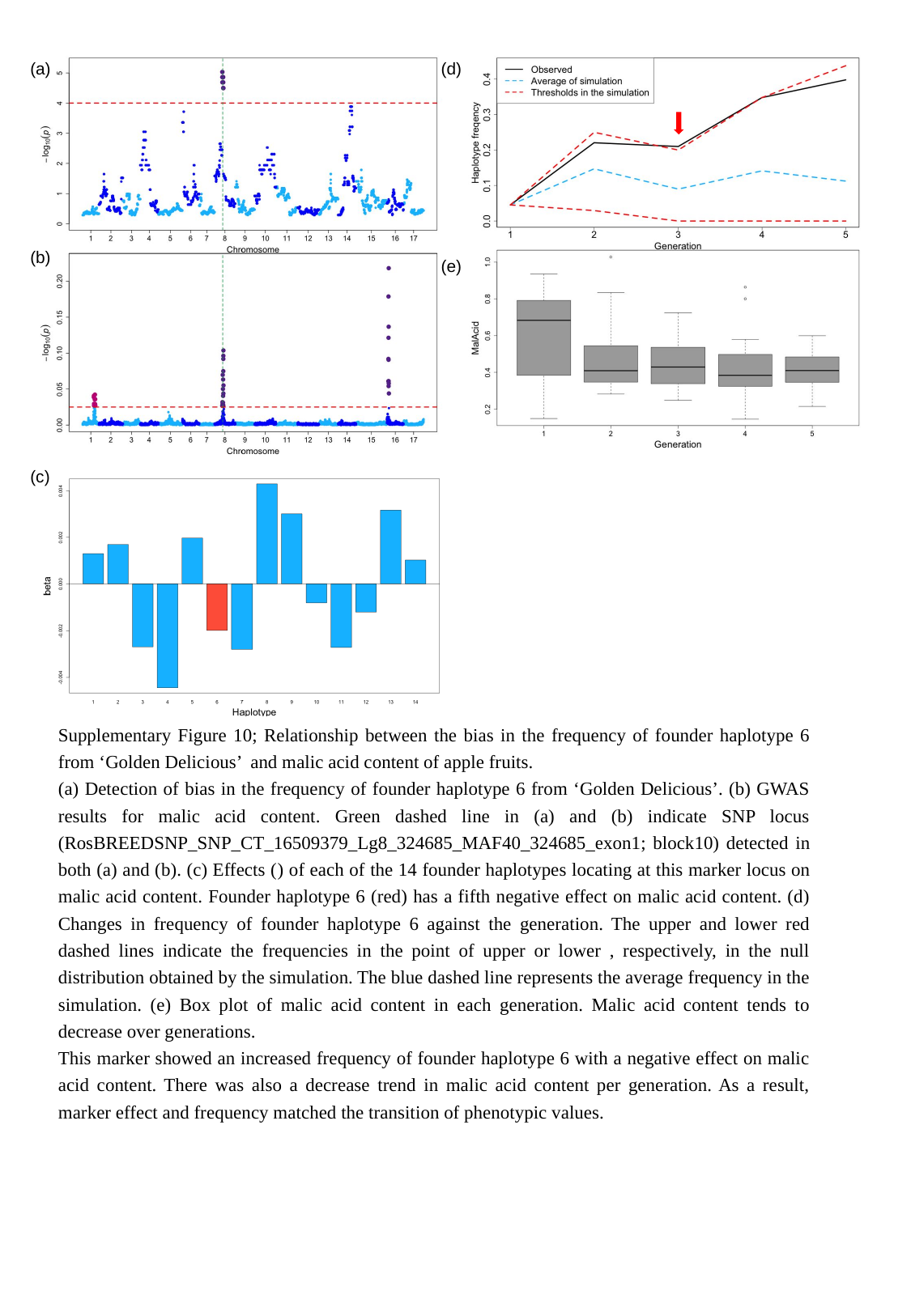

(d)
(a)
(b)
(e)
(c)

## Slide 11
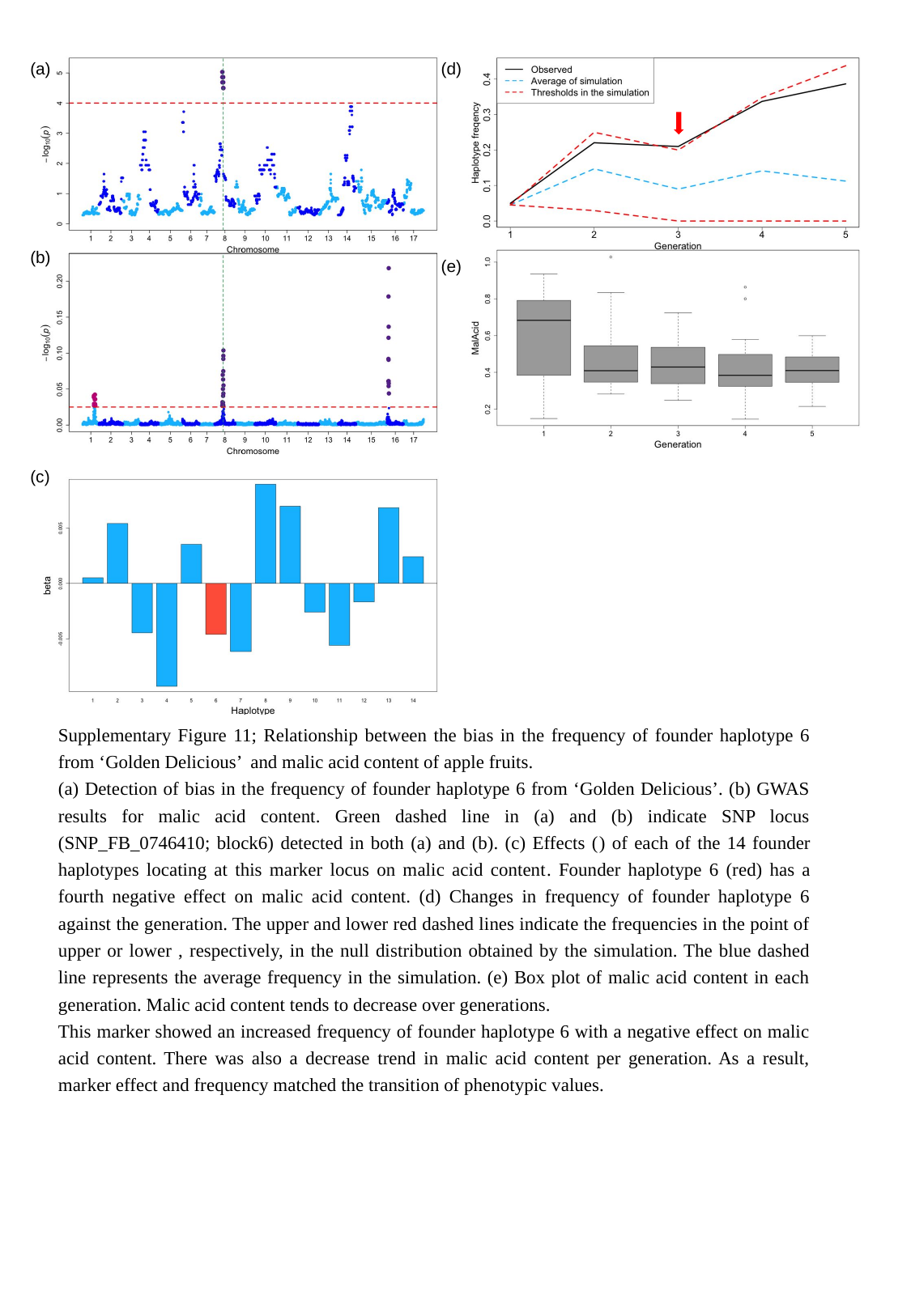

(d)
(a)
(b)
(e)
(c)

## Slide 12
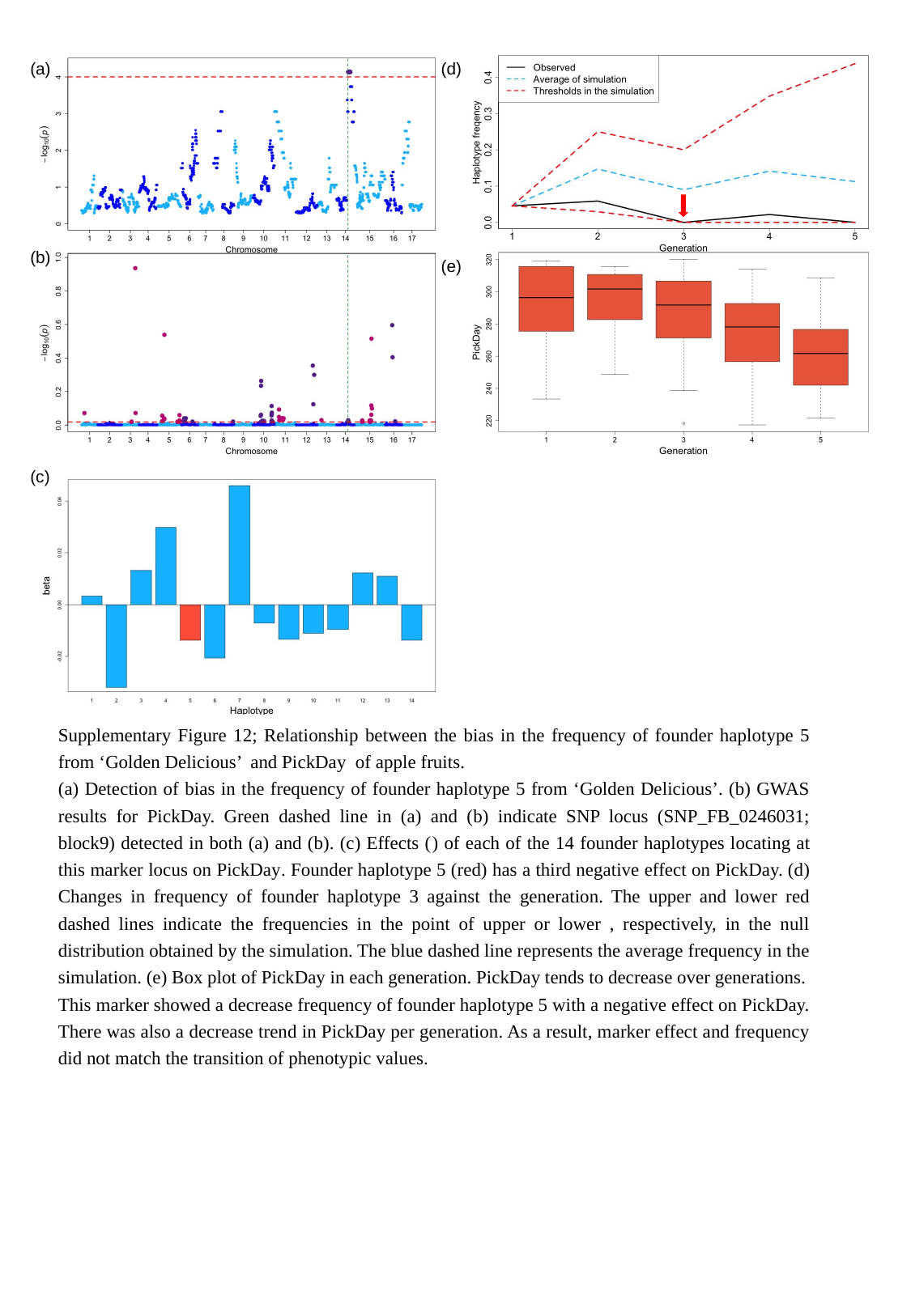

(d)
(a)
(b)
(e)
(c)

## Slide 13
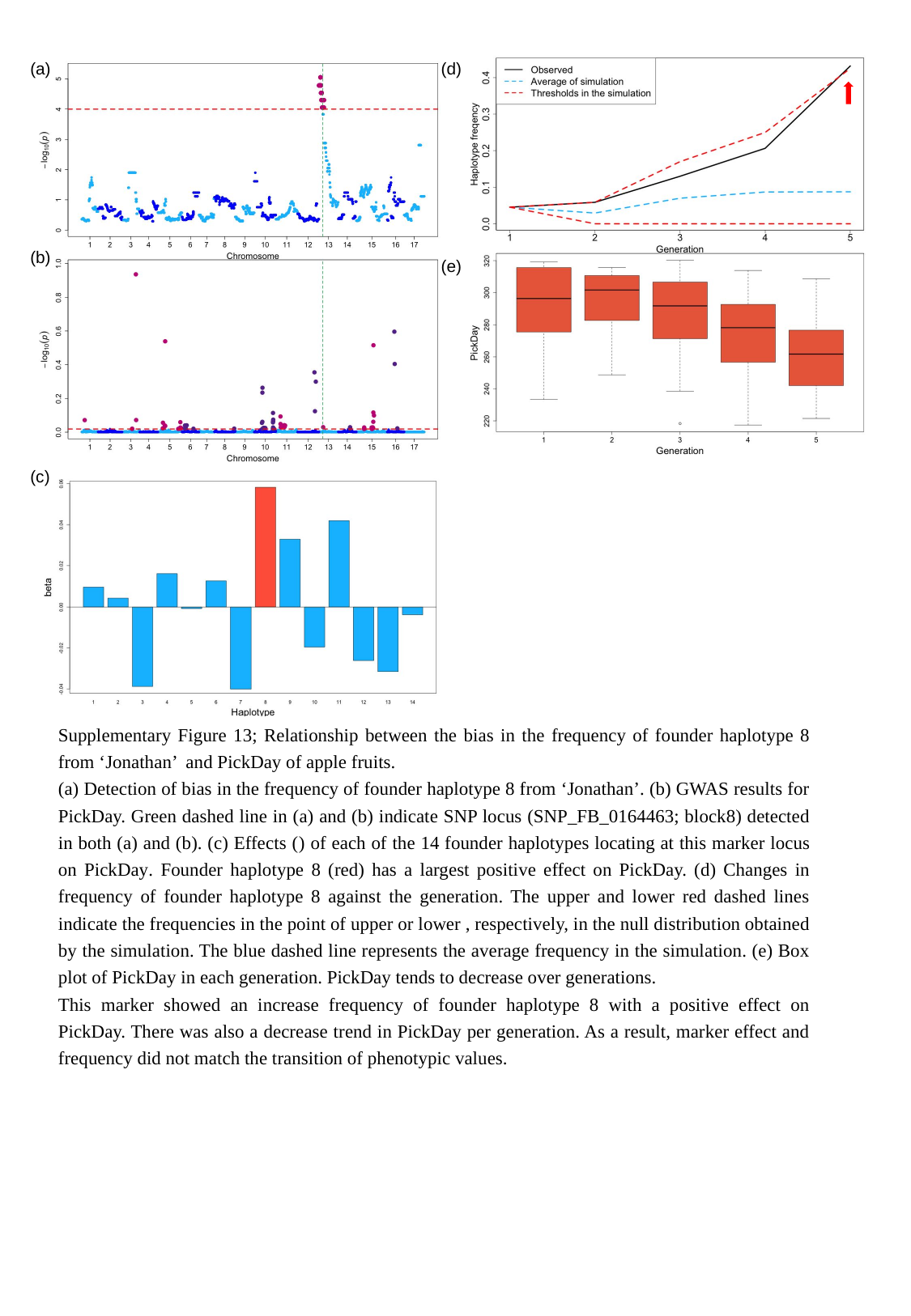

(d)
(a)
(b)
(e)
(c)

## Slide 14
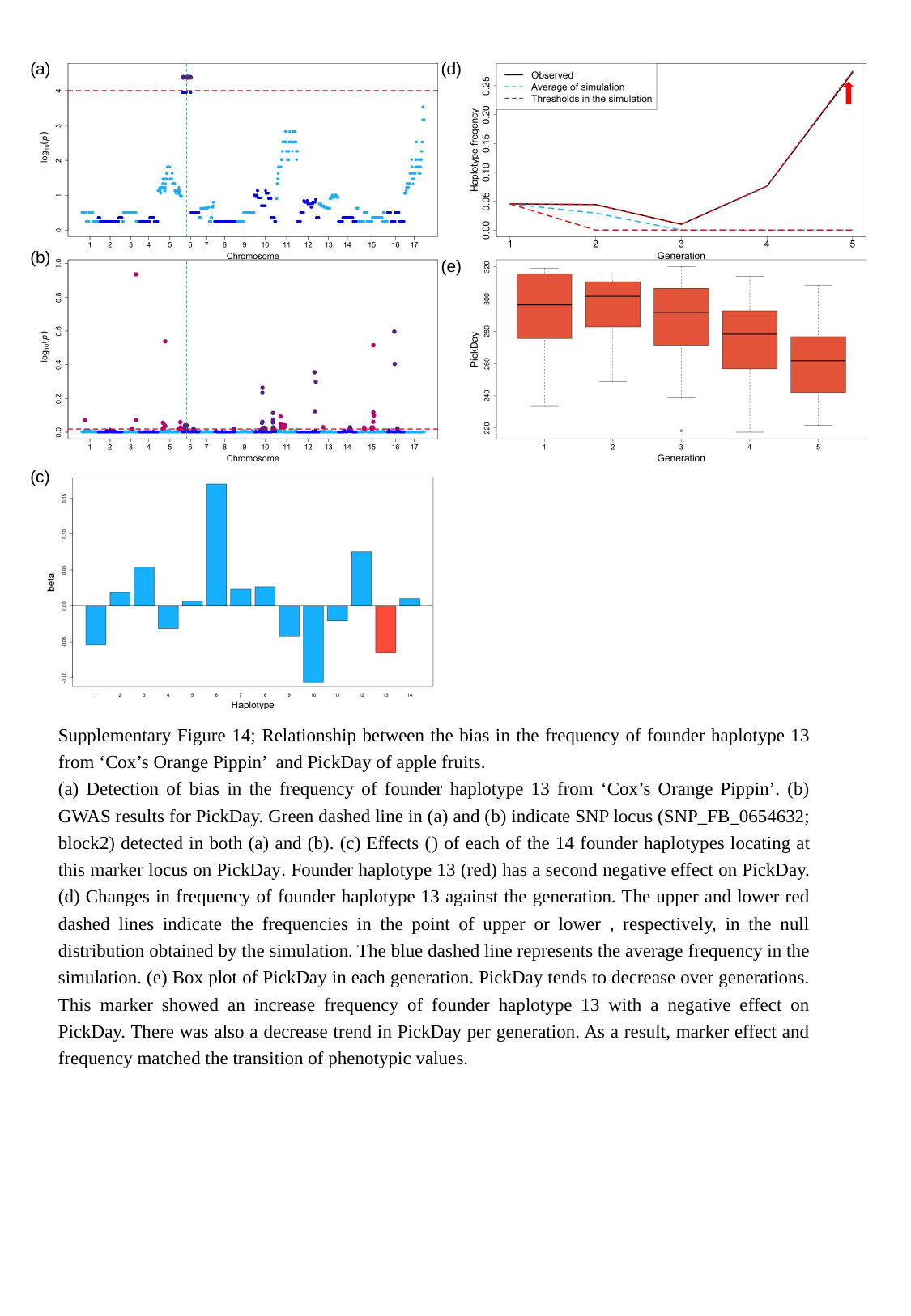

(d)
(a)
(b)
(e)
(c)

## Slide 15
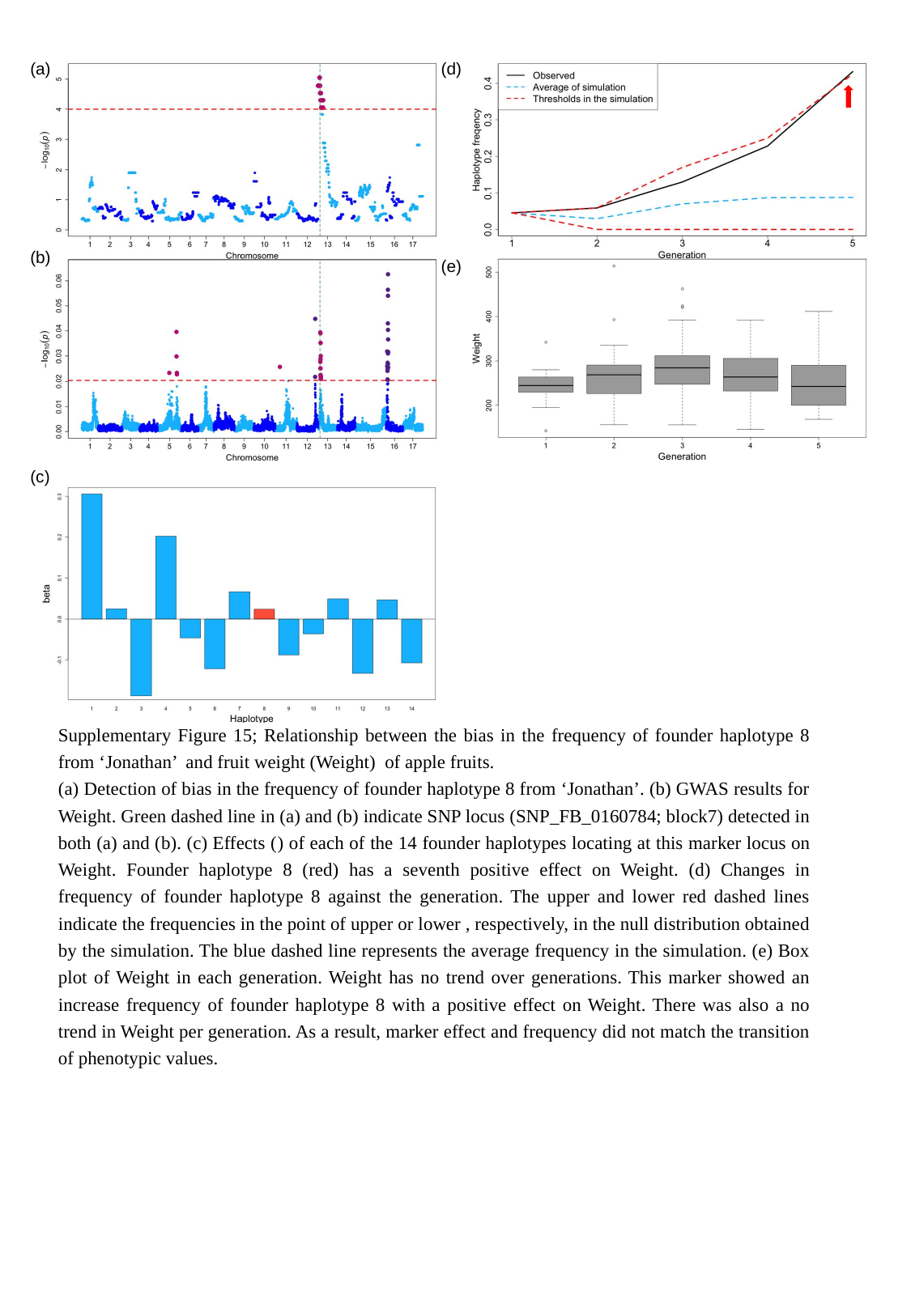

(d)
(a)
(b)
(e)
(c)

## Slide 16
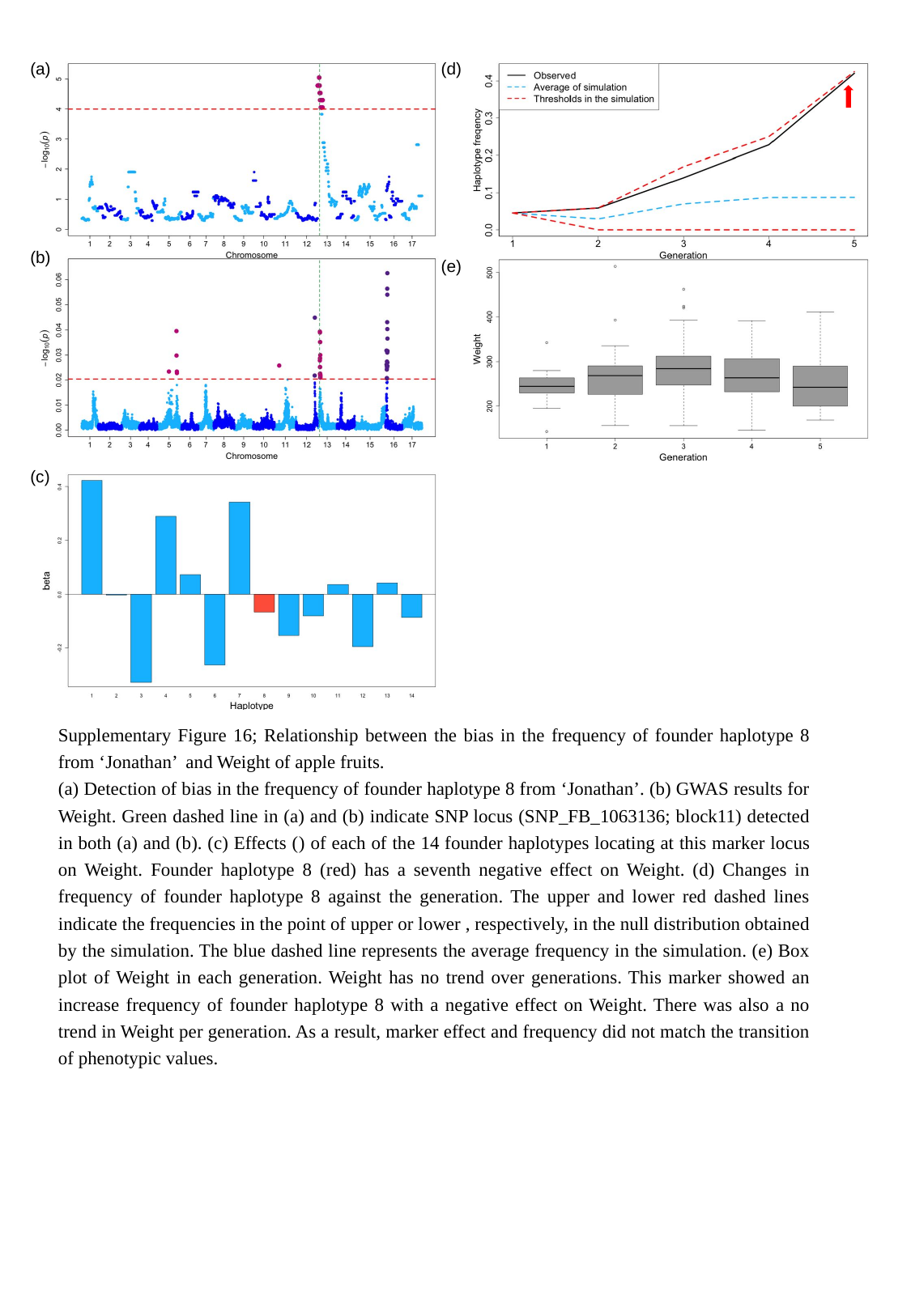

(d)
(a)
(b)
(e)
(c)
